# TCEB2 promotes M2 polarization of macrophages in triple negative breast cancer by mediating ubiquitination degradation of Slit2 through recruiting NEDD4

**DOI:** 10.1016/j.tranon.2025.102536

**Published:** 2025-09-26

**Authors:** Li Liu, Wang Xiao, Jie Zeng, Jianing Yi, Haoli Gong, Luyao Liu

**Affiliations:** a Department of Breast and Thyroid Gland Surgery, Hunan Provincial People’s Hospital and The first-affiliated hospital of Hunan normal university, Changsha 410005, Hunan Province, PR China; b Department of General Surgery, The Second Xiangya Hospital, Central South University, Changsha 410001, Hunan Province, PR China; c Department of Orthopedics, Hunan Provincial People’s Hospital and The first-affiliated hospital of Hunan normal university, Changsha 410005, Hunan Province, PR China

**Keywords:** Triple-negative breast cancer, M2 macrophage polarization, TCEB2, Ubiquitination, Slit2

## Abstract

**Background:**

M2-polarized tumor-associated macrophage infiltration is a key risk factor for poor prognosis in triple-negative breast cancer (TNBC). This study investigated the role of transcription elongation factor B polypeptide 2 (TCEB2) in regulating M2 macrophage polarization during TNBC development.

**Methods:**

The expression of gene or protein was tested by qRT-PCR, western blot, and IHC. CCK8, colony formation, wound healing, and Transwell assays were used to evaluate TNBC cell malignant behaviors, including cell viability, proliferation, migration, and invasion. The secretion levels of cytokines were detected by ELISA. Ubiquitin-based IP assays were used to detect Slit2 ubiquitination. The combined relation between TCEB2, Slit2, and NEDD4 was investigated using Co-IP assay.

**Results:**

Our results demonstrated that M2 macrophage polarization was activated in TNBC, which might be related to TCEB2 upregulation. TCEB2 knockdown reduced TNBC cell growth, migration, invasion, and their ability to induce M2 macrophage polarization. Mechanistically, TCEB2 mediated Slit2 K63 ubiquitination degradation in TNBC by interacting with NEDD4. As expected, the inhibitory effect of TCEB2 silencing on the ability of TNBC cells to induce M2 macrophage polarization was reversed by Slit2 knockdown. Finally, TCEB2 knockdown inhibited TNBC tumor growth and TNBC-induced M2 macrophage polarization *in vivo*.

**Conclusion:**

TCEB2 upregulation promoted TNBC-induced M2 macrophage polarization to accelerate TNBC development by mediating Slit2 K63-ubiquitination degradation through interacting with NEDD4.

## Highlights

• M2 macrophage polarization was activated in TNBC, which might be connected to TCEB2 overexpression.
• TCEB2 silencing inhibited TNBC cell proliferation, invasion, and their capacity to induce M2 macrophage polarization.
• TCEB2 mediated Slit2 K63 ubiquitination degradation in TNBC by recruiting NEDD4.
• TCEB2 facilitated TNBC-induced M2 macrophage polarization by regulating Slit2.
• TCEB2 knockdown inhibited TNBC-induced M2 macrophage polarization *in vivo*.

## Graphical abstract

**Figure:**
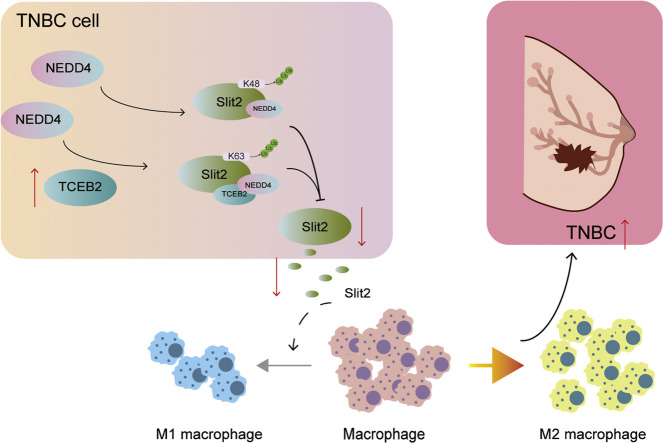

## Introduction

Triple negative breast cancer (TNBC) is a subtype of breast cancer that lacks the ER, PR, and HER-2 receptors and accounts for about 12 %−17 % of invasive breast cancer [1]. TNBC are characterized by a significant probability of visceral metastasis and high immune infiltration, resulting in high difficulty in targeted TNBC treatment and poor prognosis [2]. Due to the chemotherapy resistance caused by immunosuppression, clinical treatment for TNBC is now very difficult [3,4]. Tumor-associated macrophages (TAMs) are immune cells that infiltrate tumors and can impair anti-tumor immunity while also promoting tumor development in the tumor microenvironment (TME) [5]. The progression of TNBC is closely related to infiltration of TAMs [6,7]. In response to stimuli released by cancer cells or microenvironmental cells, TAMs can polarize into M1 or M2 macrophages, and M2-TAMs have more tumor-promoting properties [8]. As reported, M2-TAMs promote TNBC metastasis and TNBC-related deaths [4]. As a result, it is proposed that decreasing M2 macrophage polarization is a feasible therapy option for TNBC.

Transcription elongation factor B polypeptide 2 (TCEB2), also known as Elongin B (ELOB), is a core component of an E3 ubiquitin protein ligase complex that can degrade target proteins by forming complexes with various ubiquitination ligases [9]. It has been widely described that TCEB2 participates in regulating multiple malignant processes in tumor development. As evidence, TCEB2 conferred resistance to VEGF-targeted therapy in ovarian cancer [10]. Notably, TCEB2 was a differentially expressed gene in breast cancer [11]. Our preliminary research indicated that TCEB2 upregulation in TNBC was related to poor prognosis of patients. Nevertheless, the significance of TCEB2 in controlling M2 macrophage polarization during TNBC development remains unclear and warrants additional investigation.

As reported, TNBC cells can affect macrophage polarization by secreting related factors [12]. Slit guidance ligand 2 (Slit2) is a secreted protein and selectively interacts WITH Roundabout receptor 1 (Robo1) [13]. Slit2 function loss is observed in various malignancies, including breast cancer [14,15]. In addition, previous studies demonstrated that Slit2 overexpression suppressed breast tumor growth *in vivo* [16]. Notably, it was previously described that Slit2 upregulation could inhibit breast cancer metastasis by activating M1-like macrophages [17]. Therefore, it’s suggested that restoring Slit2 expression may influence TNBC-mediated macrophage polarization during TNBC progression.

Here, we found that TCEB2 facilitated the activation of M2-TAMs in TNBC by promoting Slit2 ubiquitination degradation through interacting with neural precursor cell-expressed developmentally downregulated gene 4 (NEDD4). Moreover, we discovered that TCEB2 mediated Slit2 degradation via K63-linked ubiquitination. Our research discovered the important role of TCEB2/NEDD4/Slit2 axis in regulating macrophage polarization during TNBC progression, providing a theoretical foundation for the development of innovative TNBC treatment methods.

## Materials and methods

### Clinical sample collection

40 TNBC tumor specimens and matched surrounding normal tissues were obtained post-operatively from TNBC patients at Hunan Provincial People’s Hospital and The first-affiliated hospital of Hunan normal university. In addition, a total of 40 primary breast cancer tissues (non-TNBC) were collected post-operatively from diagnosed patients. Pathology determined the patients' diagnoses, but no treatment was provided. This study was approved by the Ethics Committee of Hunan Provincial People’s Hospital and The first-affiliated hospital of Hunan normal university, and all subjects provided informed permission.

### Cell culture

Shanghai Cell Biology, Institute of the Chinese Academy of Sciences (Shanghai, China) provided the normal breast epithelial cells (MCF-10A cells), non-TNBC human breast cancer cell lines (MCF-7 and SK-BR-3), and TNBC human breast cancer cell lines (BT-549, MDA-MB-231, and MDA-MB-468 cells). THP-1 cells were purchased from ATCC (VA, USA). SK-BR-3 cells were cultured in RPMI-1640 medium (Gibco, MD, USA) containing 10 % FBS (Gibco). MDA-MB-468 cells were grown in l-15 medium (Gibco) containing 10 % FBS (Gibco). MDA-MB-231, BT-549, and MCF-7 cells were grown in DMEM (Gibco) containing 10 % 10 % FBS (Gibco). MCF-10A cells were grown in DMEM-F12 (Gibco) containing 2 mM l-glutamine, 20 ng/ml epidermal growth factor, and 10 % FBS (Gibco). THP-1 monocytes were cultured in RPMI-1640 supplemented with 10 % FBS and l-glutamine. All cells were cultured at 37 °C with 5 % CO_2_. To generate M0 macrophages, cells were seeded at 5 × 10^5^ cells/mL and treated with 100 nM phorbol 12-myristate 13-acetate (PMA; Sigma-Aldrich, MO, USA) for 48 h. Polarization into M1 and M2 phenotypes was achieved by stimulating M0 macrophages for 24 h with either 20 ng/mL IFN-γ (BioLegend, *CA*, USA) + 100 ng/mL ultrapure LPS (Sigma-Aldrich) for M1 polarization or 20 ng/mL each of IL-4 and IL-13 (PeproTech, NJ, USA) for M2 polarization.

### The co-culture of macrophages and TNBC cells

MDA-MB-231 or BT-549 cells (2  ×  10^5^) were seeded onto the top of the stratified co-culture Transwell (Corning, NY, USA) and co-cultured with macrophages (ratio 2:1) in 2 mL DMEM for 72 h. The Transwell chamber, also known as the Transwell insert, was designed to resemble a tiny cup that could be inserted into the plates. At the bottom of the cup was a permeable membrane with micropores ranging in size from 0.1 to 12.0 µm (various materials were employed according to different demands).

### Cell transfection

GenePharma (Shanghai, China) also provided the short hairpin RNAs (sh-TCEB2, sh-Slit2, and sh-NEDD4), the overexpression plasmid (OE-TCEB2), and their negative controls. The vectors were introduced into cells using Lipofectamine 3000 (Invitrogen, *CA*, USA).

### Cell counting kit-8 (CCK-8) assay

Cells were cultured in 96-well plates (2 × 10^3^ cells/well) for 24 h and incubated for 3 h with 10 μL CCK-8 solution (Yeason, Shanghai, China) at 37 °C for 3 h. The absorbance at 450 nm was measured.

### Colony formation assay

Cells were cultured in six-well plates at a density of 1 × 10^3^ cells/well for 1 week. The colonies were fixed with methanol for 10 min and stained with crystal violet (1 %) for 5 min. The number of colonies was counted using an Olympus microscope (Tokyo, Japan).

### Wound healing assay

Cells were grown in 6-well plates (5 × 10^5^ cells/well) for 12 h. A sterile pipette tip was used to construct an artificial wound following removal of the media. Cells were washed, and the images were captured at 0 h and 24 h.

### Transwell invasion assay

DMEM (500 μL) containing 1 × 10^4^ cells was added to the upper chamber precoated with Matrigel (BD, NJ, USA), and complete DMEM (1000 μL) was added to the bottom chamber. Cells on the top chamber were removed after 12 h, while cells on the bottom chamber were fixed and stained with 0.5 % crystal violet. An Olympus microscope was used to image cells.

### Quantitative real-time polymerase chain reaction (qRT-PCR)

Total RNA was extracted using TRIzol (Invitrogen). cDNA synthesis was carried out using a reverse transcription kit (ThermoFisher Scientific, MA, USA). ThermoFisher Scientific's SYBR was used for qRT-PCR. GAPDH was employed as the reference gene. The data were analyzed using 2^−ΔΔCT^ method. The data were analyzed using 2^−ΔΔCT^ method. The primers used in the study were listed as follows (5′−3′):

TCEB2 (F): CGAACTGAAGCGCATCGTC
TCEB2 (R): TCCAAGAGTTGGTCATCCTTGT
NEDD4 (F): TCAGGACAACCTAACAGATGCT
NEDD4 (R): TTCTGCAAGATGAGTTGGAACAT
Slit2 (F): GCGAAGCTATACAGGCTTGAT
Slit2 (R): TGCAGTCGAAAAGTCCTAAGTTT
iNOS (F): TGGAGCCAGTTGTGGATTGTC
iNOS (R): GGTCGTAATGTCCAGGAAGTAG
IL-23 (F): TTATGAGAAGCTGCTAGGATCG
IL-23 (R): GAAGGATTTTGAAGCGGAGAAG
IL-1β (F): TGGGAAACAACAGTGGTCAGG
IL-1β (R): CCATCAGAGGCAAGGAGGAA
Arg1 (F): GTGGAAACTTGCATGGACAAC
Arg1 (R): AATCCTGGCACATCGGGAATC
VEGF (F): AGGGCAGAATCATCACGAAGT
VEGF (R): AGGGTCTCGATTGGATGGCA
IL-10 (F): TTGCTGGAGGACTTTAAGGGT
IL-10 (R): CTTGATGTCTGGGTCTTGGTT
GAPDH (F): AGGTCGGTGTGAACGGATTTG
GAPDH (R): GGGGTCGTTGATGGCAACA

### Flow cytometry

Cell concentration was diluted to 1 × 10^7^/mL using PBS containing 10 % FBS. Cells were then stained for 30 min with anti-CD80 (Abcam, Cambridge, UK, 1:500, ab134120) and anti-CD163 (Abcam, 1:500, ab182422) in the dark. Following two washes, cells were suspended in 2 mL PBS and detected using flow cytometry (BD, NJ, USA).

### Enzyme-linked immunosorbent assay (ELISA)

iNOS, IL-23, IL-1β, Arg1, VEGF, IL-10, and Slit2 levels were detected by the human iNOS ELISA kit (Abcam, ab253217), the human IL-23 ELISA kit (Abcam, ab221837), the human IL-1β ELISA kit (ThermoFisher Scientific, BMS224–2), the human Arg1 ELISA kit (Abcam, ab230930), the human VEGF ELISA kit (Abcam, ab222510), the human IL-10 ELISA kit (ThermoFisher Scientific, BMS215–2), and the human Slit2 ELISA kit (Jianglai Biotech, Shanghai, China, JL43722–96T), respectively, according to the manuals. The OD values were recorded at 450 nm and analyzed by Origin 9.5 software.

### Coimmunoprecipitation (Co-IP)

MDA-MB-231 and BT-549 cells were lysed in lysis buffer and incubated with Sepharose CL-4B bead-conjugated IgG (Abcam, 1:50, ab172730), TCEB2 (Abcam, 1:50, ab168836), Slit2 (Abcam, 1:50, ab134166), or NEDD4 (Abcam, 1:50, ab240753) antibody for 4 h. The bound proteins were eluted and assessed using western blot.

### Western blot analysis

A BCA kit (ThermoFisher Scientific) was used to measure the proteins after they had been separated using RIPA (Beyotime, Shanghai, China). The total protein was isolated by 10 % SDS-PAGE and transferred to a Millipore PVDF membrane (MA, USA). The membranes were blocked and incubated overnight with antibodies against TCEB2 (ab168836), Slit2 (ab246503), NEDD4 (ab240753), iNOS (ab178945), CD80 (ab134120), Arg1 (ab133543), CD163 (ab182422), and GAPDH (ab8245). The membranes were then hybridized with the secondary antibody (ab7090) for 60 min. The protein bands were visualized by ECL (Beyotime) and quantified by Image J. All antibodies were purchased from Abcam (Cambridge, UK) and diluted according to the instructions.

### Cycloheximide (CHX) chase assay

MDA-MB-231 and BT-549 cells were transfected with sh-NC or sh-TECB2, then incubated with 100 μg/mL CHX (MedChemExpress, NJ, USA) for 0, 2, 4, and 8 h. Slit2 level was determined using western blot.

### Analysis of Slit2 ubiquitination

Tumor tissues and cells underwent lysis with 1 % SDS buffer and boiling (10 min). After centrifugation, the supernatants were diluted with NP-40 lysis buffer and were incubated for 12 h with 30 µL of protein A/G IP magnetic bead-conjugated IgG (Abcam, 1:100, ab172730) or Slit2 antibody (Abcam, 1:50, ab7665). The beads were washed, and the bead-associated proteins were isolated and detected by western blot with anti-Ub (Abcam, 1:1000, ab140601), anti-Ub-K48 (Abcam, 1:1000, ab140601), and anti-Ub-K63 (Abcam, 1:1000, ab179434) antibodies.

### Animal experiments

SJA LABORATORY provided 10 female BALB/c nude mice (4–5-week-old). After acclimation for 1 week, mice were randomly classified into two groups: sh-NC and sh-TCEB2 (with 5 mice in each group). BALB/C nude mice were subcutaneously injected with MDA-MB-231 cells (0.1 mL PBS, 2 × 10^6^ cells), which were stably transfected with sh-NC or sh-TCEB2. The tumor volume was measured every 5 d according to the formula: *V* = lw^2^/2. The mice were then euthanized 25 d after injection, and the tumor tissues were collected. Hunan Provincial People’s Hospital and The first-affiliated hospital of Hunan normal university approved the animal studies.

### Immunohistochemistry (IHC)

The tumor sections (4 μm in thickness) were then rehydrated and microwaved in sodium citrate buffer to retrieve antigens. The sections were subsequently blocked and incubated overnight with antibodies against Ki67 (Abcam, 1:200, ab15580), CD80 (Abcam, 1:150, ab254579), and CD163 (Abcam, 1:500, ab182422), followed by 1 h incubation with an appropriate secondary antibody (Abcam, 1:500, ab150077). DAB was used to stain the sections, which were subsequently counterstained with hematoxylin before being dehydrated and mounted. The photographs were obtained with an Olympus microscope. The positive rate was analyzed using ImageJ.

### Data analysis

All data were obtained from three separate trials. The statistical data, which were reported as Mean±SD, were examined using Graphpad Prism 7.0. Tumors were divided according to expression (median cutoff), and differences in clinicopathological features were compared using chi-squared analysis. The differences between the two groups were investigated using Student’s *t*-tests. One-way ANOVA was performed to compare differences between groups. Cumulative survival was evaluated using the Kaplan-Meier method (log-rank test). The *p*-values <0.05 were regarded as significant.

## Results

### The polarization of M2 macrophages was higher in TNBC, which might be related to TCEB2 upregulation

**Fig. 1:**
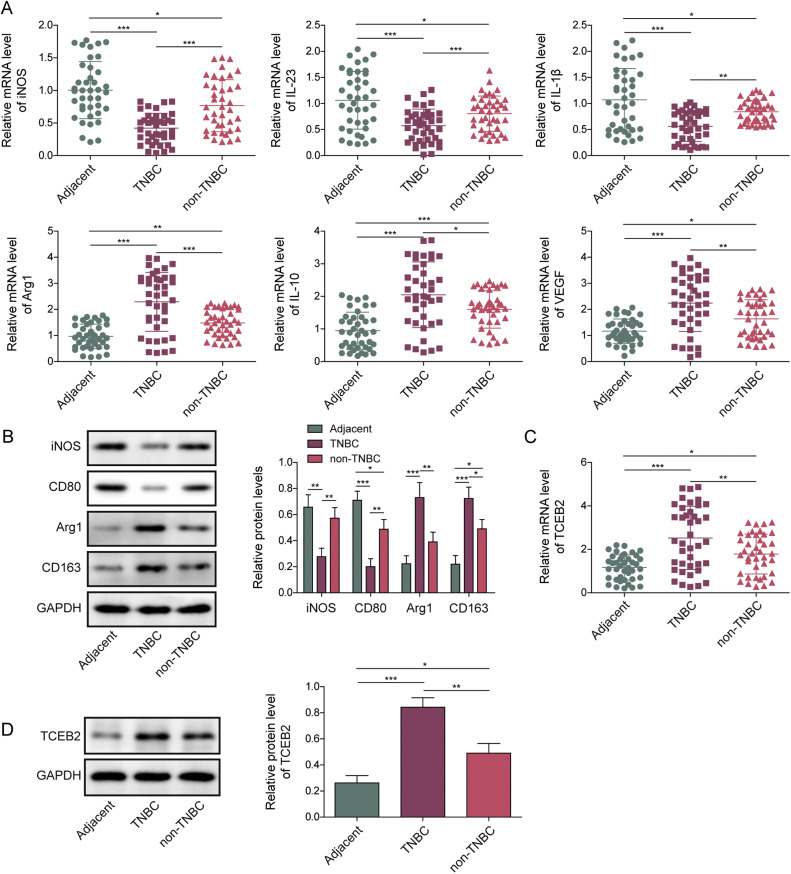

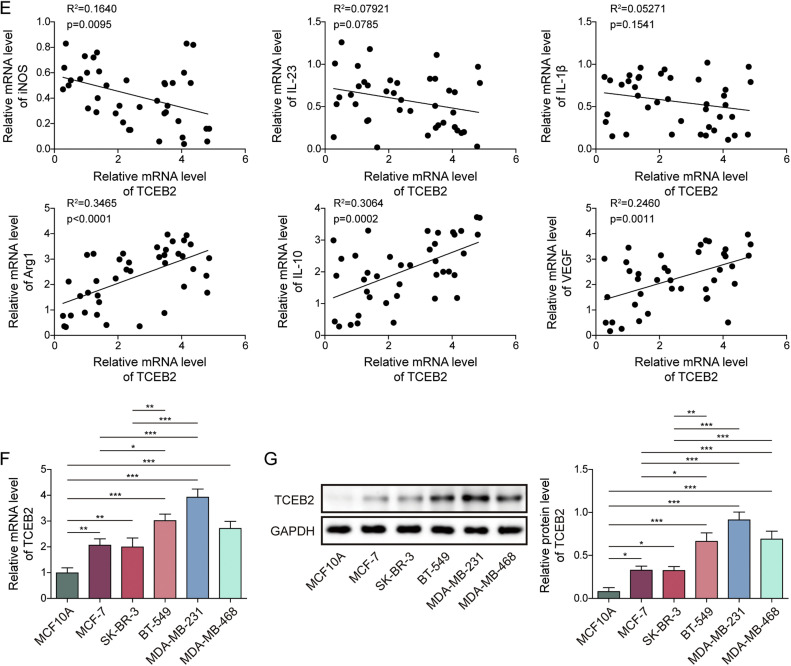
**The polarization of M2 macrophages was higher in TNBC, which might be related to TCEB2 upregulation**. TNBC tumor tissues and primary breast cancer tissues (non-TNBC), as well as paired adjacent normal tissues, were collected. (A) The mRNA levels of iNOS, IL-23, IL-1β, Arg1, VEGF, and IL-10 in tissues were determined by qRT-PCR(*N* = 40). (B) Western blot was employed to detect iNOS, CD80, Arg1, and CD163 protein levels in tissues (*N* = 40). (C-D) The mRNA and protein levels of TCEB2 were determined by qRT-PCR and western blot, respectively (*N* = 40). (E) The correlation between TCEB2 expression and the expressions of M2 macrophage cytokines (Arg1, VEGF, and IL-10), and the correlation between TCEB2 expression and the expressions of M1 macrophage cytokines (iNOS, IL-23, and IL-1β) were analyzed by Pearson correlation analysis (*N* = 40). (F-G) The mRNA and protein levels of TCEB2 in normal breast epithelial cells (MCF-10A cells) and human breast cancer cell lines (MCF-7, SK-BR-3, BT-549, MDA-MB-231, and MDA-MB-468 cells) were measured by qRT-PCR and western blot, respectively (*N* = 3). The measurement data were presented as mean ± SD. *p < 0.05, **p < 0.01, ***p < 0.001.

40 TNBC tumor specimens and matched surrounding normal tissues were obtained post-operatively from TNBC patients. Our analysis revealed that high expression of TCEB2 and NEDD4 was significantly associated with more aggressive tumor stages, suggesting their potential roles in disease progression. In contrast, high Slit2 expression correlated with earlier disease stages and supporting its protective role (**Table S1**). As displayed in Fig. 1**A**, the mRNA levels of M1 macrophage cytokines (iNOS, IL-23, and IL-1β) in breast cancer tissues were significantly reduced, and the mRNA levels of M2 macrophage cytokines (Arg1, VEGF, and IL-10) were elevated compared with precancerous tissues, and these changes were more significant in TNBC tissues. Similar trends were observed in the protein levels of M1 markers (iNOS and CD80) and M2 markers (Arg1 and CD163) (Fig. 1**B**). It also turned out that TCEB2 was overexpressed in breast cancer tissues compared to paracancerous tissues, and this upregulation was more significant in TNBC tissues (Fig. 1**C and D**). In addition, TCEB2 expression was shown to be favorably connected with M2 macrophage cytokine expressions (Arg1, VEGF, and IL-10) but negatively correlated with M1 macrophage cytokine expressions (iNOS and IL-23) (Fig. 1**E**). Moreover, TCEB2 was overexpressed in breast cancer cells (non-TNBC) (MCF-7 and SK-BR-3 cells) compared to normal breast epithelial cells (MCF-10A cells), and its upregulation was more significantly in TNBC cells (BT-549, MDA-MB-231, and MDA-MB-468 cells) (Fig. 1**F and G**). Collectively, TCEB2 upregulation in TNBC was related to high M2 macrophage polarization.

### TCEB2 enhanced TNBC cell proliferation and invasion while promoting TNBC-induced M2 macrophage polarization

**Fig. 2:**
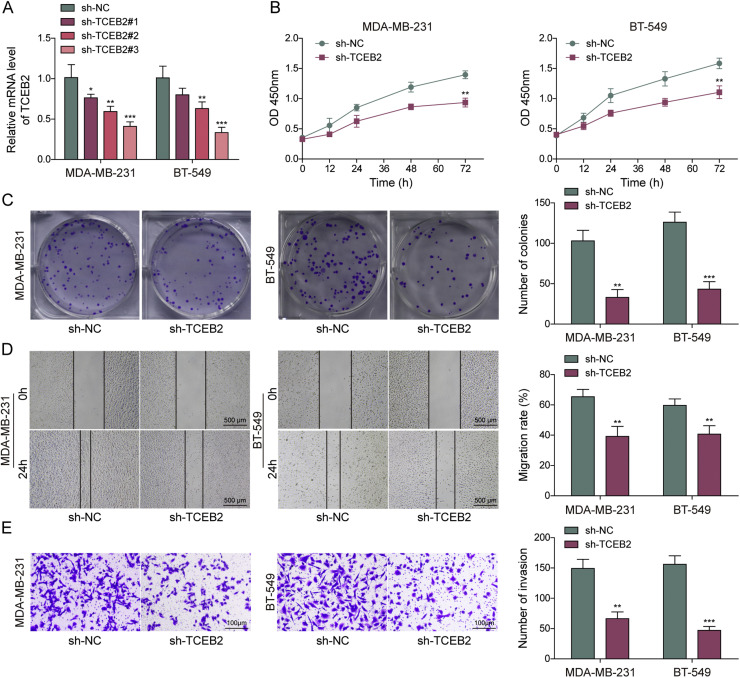

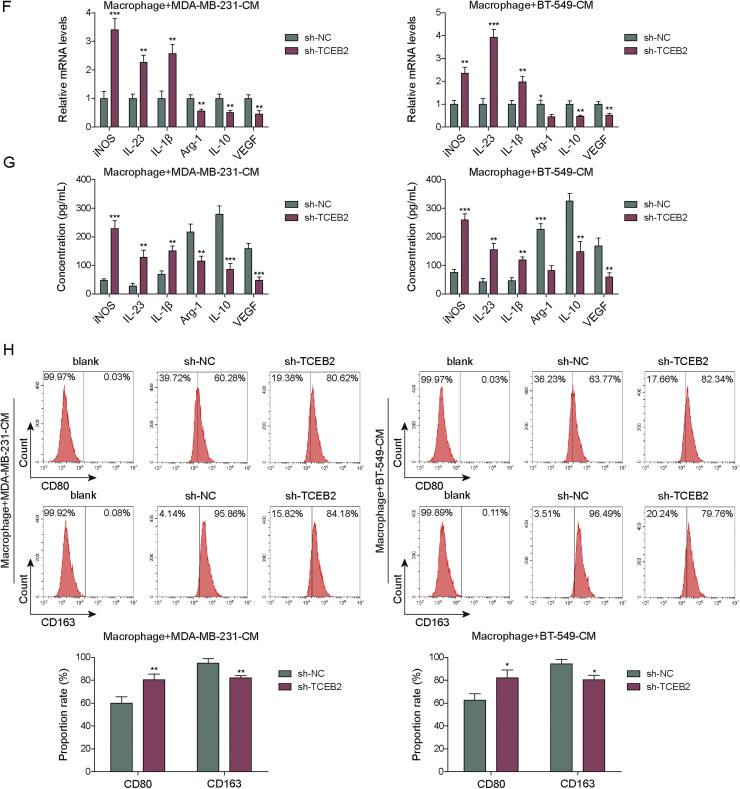
**TCEB2 enhanced TNBC cell proliferation and invasion and promoted TNBC-induced M2 macrophage polarization**. (A) qRT-PCR was performed to examine TCEB2 mRNA level in MDA-MB-231 and BT-549 cells following sh-NC, sh-TCEB2#1, sh-TCEB2#2, or sh-TCEB2#3 transfection. MDA-MB-231 and BT-549 cells were transfected with sh-NC or sh-TCEB2#3. (B-C) Cell viability and proliferation were analyzed by CCK8 assay and colony formation assay, respectively. (D-E) Cell migration and invasion were determined by wound healing assay and Transwell assay, respectively. THP-1 cells were incubated with 100 ng/mL PMA for 24 h to obtain macrophages, and macrophages were then co-cultured with TNBC cells. (F-G) qRT-PCR and ELISA were adopted to measure the mRNA and secretion levels of iNOS, IL-23, IL-1β, Arg1, VEGF, and IL-10 in macrophages. (H) CD163 and CD80 levels in macrophages were analyzed by flow cytometry. The measurement data were presented as mean ± SD. *N* = 3. *p < 0.05, **p < 0.01, ***p < 0.001.

To study the significance of TCEB2 in controlling M2 macrophage polarization during TNBC progression, TCEB2 knockdown was induced in MDA-MB-231 and BT-549 cells. Both sh-TCEB2–1, sh-TCEB2–2, and sh-TCEB2–3 transfections significantly reduced TCEB2 expression level in cells (Fig. 2**A**). The knockdown efficiency of sh-TCEB2–3 was observed to be higher (Fig. 2**A**), so sh-TCEB2–3 was chosen for following investigations. It was subsequently shown that TCEB2 silencing markedly inhibited TNBC cell viability and proliferation (Fig. 2**B and C**). Additionally, TNBC cell migration and invasion were remarkably suppressed by TCEB2 knockdown (Fig. 2**D and E**). TNBC cells were then co-cultured with macrophages. Initial characterization confirmed successful differentiation of THP-1 cells into M0 macrophages, with morphological changes apparent by 48 h post-PMA treatment (**Fig. S1A**). Flow cytometry analysis demonstrated that TNBC-conditioned macrophage cultures contained a distinct CD80^+^CD163^+^ population that was virtually absent in M0 controls and significantly different from classical M1 or M2 populations (**Fig. S1B**). This suggests TNBC microenvironment uniquely induces a hybrid macrophage phenotype rather than simply shifting cells toward classical M1 or M2 states. It also turned out that TCEB2 knockdown in TNBC cells repressed TNBC-induced M2 macrophage polarization (Fig. 2**F and G**). Consistently, TCEB2 knockdown in TNBC cells markedly increased CD80 proportion rate in macrophages and reduced CD163 proportion rate (Fig. 2**H**). Taken together, TCEB2 knockdown inhibited TNBC cell proliferation, migration, and invasion, and TNBC-induced M2 macrophage polarization.

### TCEB2-mediated Slit2 ubiquitination degradation in TNBC

**Fig. 3:**
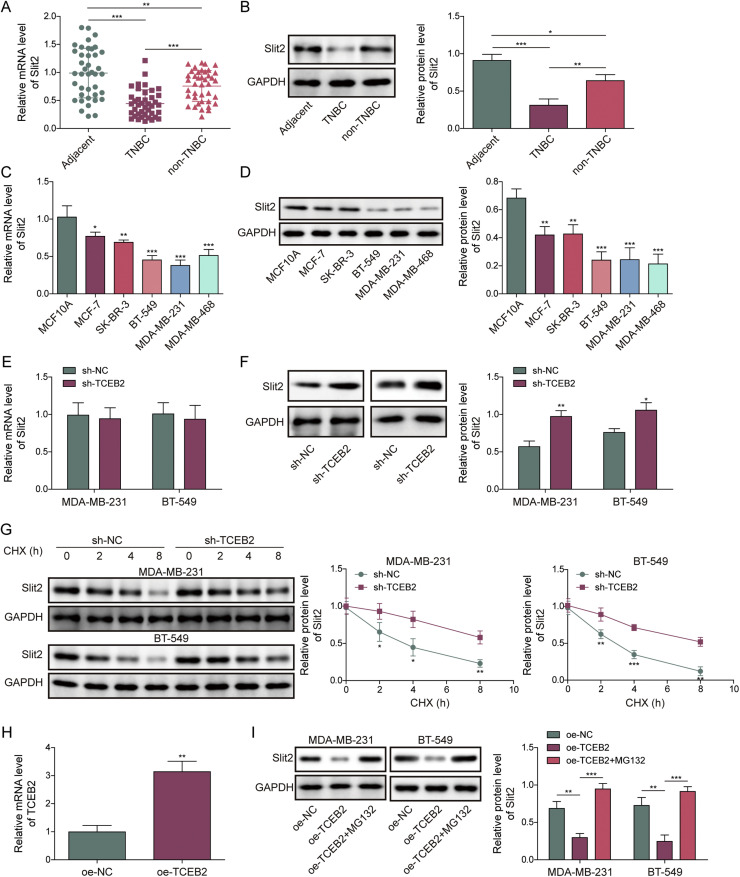

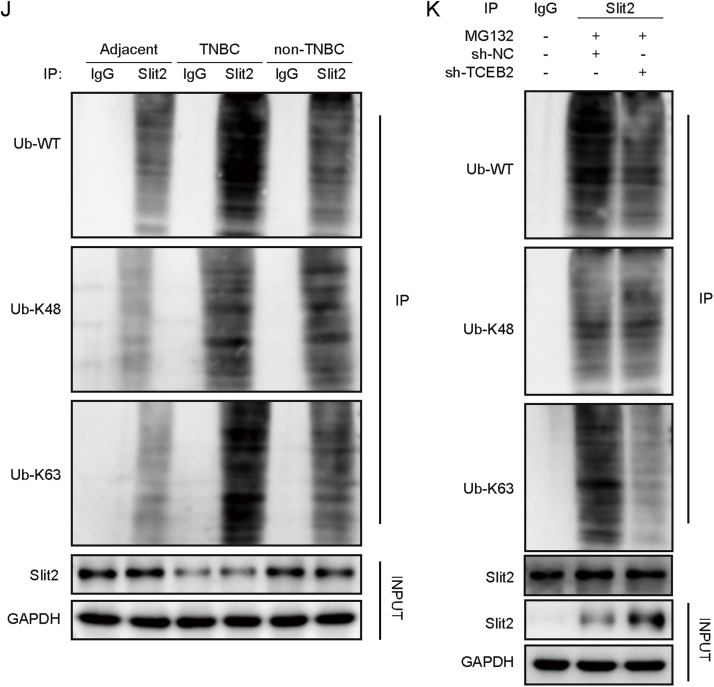
**TCEB2 mediated Slit2 ubiquitination degradation in TNBC**. (A-B) The mRNA and protein levels of Slit2 in tissues were measured by qRT-PCR and western blot, respectively. (C-D) The mRNA and protein levels of Slit2 in normal breast epithelial cells (MCF-10A cells) and human breast cancer cell lines (MCF-7, SK-BR-3, BT-549, MDA-MB-231, and MDA-MB-468 cells) were measured by qRT-PCR and western blot, respectively. (E-F) The mRNA and protein levels of Slit2 in MDA-MB-231 and BT-549 cells after sh-NC or sh-TCEB2 transfection were measured by qRT-PCR and western blot, respectively. (G) Slit2 protein degradation in TNBC cells after sh-NC or sh-TCEB2 transfection was detected by CHX chase assay. (H) TCEB2 mRNA level in MDA-MB-231 and BT-549 cells following OE-NC or OE-TCEB2 transfection was examined by qRT-PCR. (I) Western blot was performed to detect Slit2 protein in MDA-MB-231 and BT-549 cells following OE-NC or OE-TCEB2 transfection combined with MG132 treatment. (J) Slit2 ubiquitination level in tissues was measured by Slit2 ubiquitination analysis. (K) Slit2 ubiquitination level in MDA-MB-231 and BT-549 cells following sh-NC or sh-TCEB2 transfection combined with MG132 treatment was detected using Slit2 ubiquitination analysis. The measurement data were presented as mean ± SD. *N* = 3. *p < 0.05, **p < 0.01, ***p < 0.001.

As revealed in Fig. 3**A and B**, Slit2 was lowly expressed in breast cancer tissues compared with precancerous tissues, and this downregulation was more significant in TNBC tissues. A similar trend was observed in Slit2 expression levels in TNBC cells (Fig. 3**C and D**). It was found that TCEB2 knockdown significantly elevated Slit2 protein level in TNBC cells but didn’t affect Slit2 mRNA level significantly (Fig. 3**E and F**). Considering that TCEB2 is a core component of an E3 ubiquitin protein ligase complex [9], it’s speculated that TCEB2 might mediate Slit2 ubiquitination degradation in TNBC. As shown in Fig. 3**G**, TCEB2 knockdown reduced Slit2 protein degradation in TNBC cells. OE-TCEB2 transfection significantly increased TCEB2 mRNA level in TNBC cells (Fig. 3**H**). Furthermore, TCEB2 overexpression lowered Slit2 protein level in TNBC cells, which was reversed by MG132 (Proteasome inhibitor) treatment (Fig. 3**I**). It also turned out that Slit2 was ubiquitinated and degraded in breast cancer tissues compared with precancerous tissues (mainly K63 ubiquitination), and this change was more significant in TNBC tissues (Fig. 3**J**). Moreover, TCEB2 knockdown markedly reduced Slit2 protein K63 ubiquitination level in TNBC cells (Fig. 3**K**). In summary, Slit2 was ubiquitinated and degraded in TNBC, which was mediated by TCEB2.

### TCEB2 mediated Slit2 K63-ubiquitination degradation in TNBC by recruiting NEDD4

**Fig. 4:**
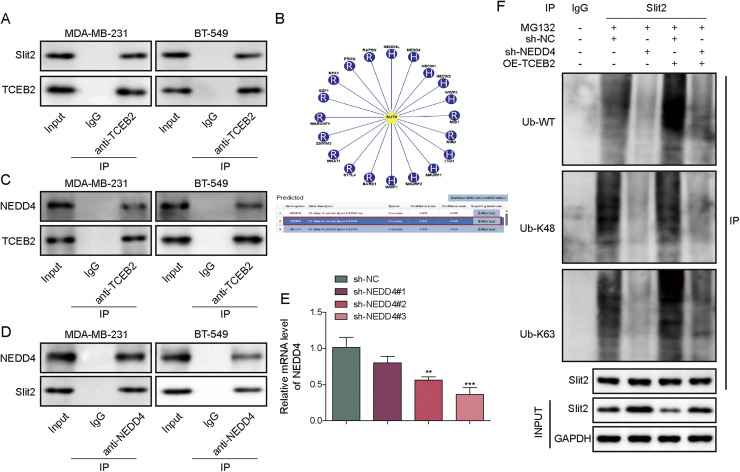
**TCEB2 mediated Slit2 K63-ubiquitination degradation in TNBC by recruiting NEDD4**. (A) The interaction between TCEB2 and Slit2 in MDA-MB-231 or BT-549 cells was analyzed by Co-IP assay. (B) The E3 ubiquitination ligases acting on Slit2 were predicted using bioinformatics. (C-D) Co-IP assay was employed to analyze the interaction between NEDD4 and Slit2 and the interaction between NEDD4 and TCEB2. (E) qRT-PCR was performed to examine NEDD4 mRNA level in MDA-MB-231 and BT-549 cells following sh-NC, sh-NEDD4#1, sh-NEDD4#2, or sh-NEDD4#3 transfection. (F) MDA-MB-231 and BT-549 cells were co-transfected with sh-NEDD4 and OE-TCEB2, and Slit2 ubiquitination level in cells was detected using Slit2 ubiquitination analysis. The measurement data were presented as mean ± SD. *N* = 3. **p < 0.01, ***p < 0.001.

As revealed by CoIP assay, TCEB2 directly bound to Slit2 in TNBC cells (Fig. 4**A**). As reported, NEDD4 can enhance polyubiquitination by interacting with complexes dominated by TCEB2 [18]. Herein, as predicted by bioinformatics, NEDD4 could participate in the ubiquitination of Slit2 with a high confidence level (Fig. 4**B**). It was subsequently confirmed that NEDD4 directly bound with both Slit2 and TCEB2 (Fig. 4**C-D**). NEDD4 knockdown was induced in MDA-MB-231 and BT-549 cells. Both sh-NEDD4–1, sh-NEDD4–2, and sh-NEDD4–3 transfections significantly reduced NEDD4 expression level in TNBC cells (Fig. 4**E**). The knockdown efficiency of sh-NEDD4–3 was observed to be higher (Fig. 4**E**), so sh-NEDD4–3 was selected for subsequent experiments. It was observed that both the total ubiquitination level, K63 ubiquitination level and K48 ubiquitination level of Slit2 protein in TNBC cells were reduced by NEDD4 knockdown, while TCEB2 overexpression increased Slit2 protein K63 ubiquitination level (Fig. 4**F**). Meanwhile, sh-NEDD4 and OE-TCEB2 co-transfection reduced the overall ubiquitination level of Slit2 protein in TNBC cells (Fig. 4**F**). Collectively, TCEB2 promoted Slit2 K63-ubiquitination degradation in TNBC by interacting with NEDD4.

### TCEB2 promoted TNBC-induced M2 macrophage polarization by acting on Slit2

To demonstrate the importance of the key indicator Slit2 in regulating macrophage polarization during TNBC development, M0 macrophages were treated with 10 μg/mL exogenous recombinant Slit2 protein for 2 h. As shown in **Fig. S2A**, the treatment of exogenous recombinant Slit2 protein significantly increased Sli2 in the medium. In addition, the treatment of exogenous recombinant Slit2 protein significantly promoted M2 macrophage polarization (**Fig. S2B-C**). Furthermore, the treatment of exogenous recombinant Slit2 protein markedly increased CD80/CD163 rate (**Fig. S2D**). To study the role of TCEB2 and Slit2 in controlling M2 macrophage polarization during TNBC progression, both TCEB2 knockdown and Slit2 knockdown were induced in TNBC cells. As demonstrated in Fig. 5**A and B**, both sh-Slit2–1, sh-Slit2–2, and sh-Slit2–3 transfection significantly reduced Slit2 expression level in MDA-MB-231 and BT-549 cells. The knockdown efficiency of sh-Slit2–3 was observed to be higher (Fig. 5**A and B**), hence it was chosen for following investigations. It was subsequently displayed that Slit2 downregulation prevented sh-TCEB2-induced inhibition on TNBC cell viability and proliferation (Fig. 5**C and D**). Meanwhile, Slit2 silencing restored the inhibitory impacts of TCEB2 knockdown on TNBC cell migration and invasion (Fig. 5**E and F**). It was also observed that TCEB2 knockdown Slit2 secretion, while Slit2 silencing reversed this effect (Fig. 5**G**). In addition, the inhibitory impact of TCEB2 silencing on TNBC-induced M2 macrophage polarization was weakened by Slit2 silencing (Fig. 5**H and I**). Moreover, TCEB2 knockdown in TNBC cells markedly increased CD80 proportion rate and reduced CD163 proportion rate in co-cultured macrophages, while this change was eliminated by Slit2 silencing (Fig. 5**J**). In conclusion, TCEB2 facilitated TNBC-induced M2 macrophage polarization by regulating Slit2.

**Fig.**
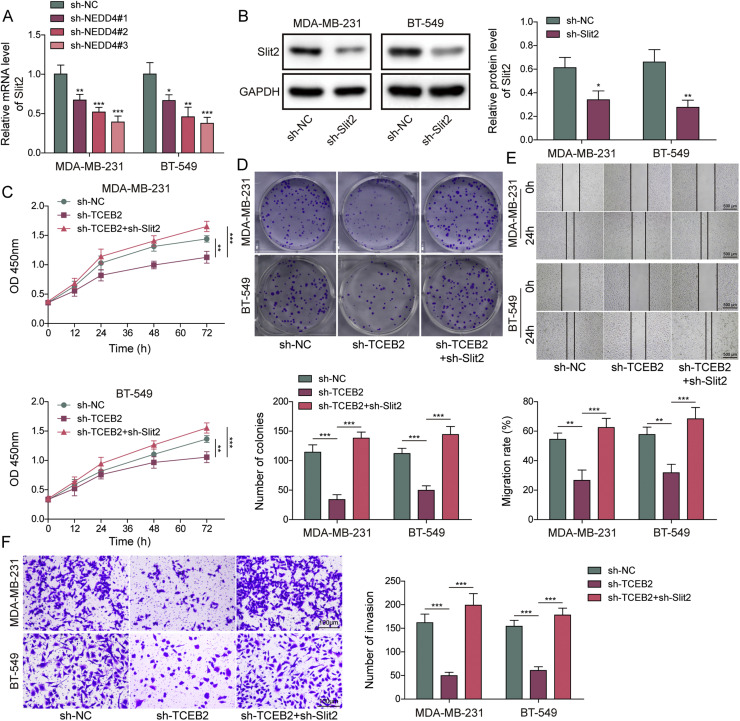

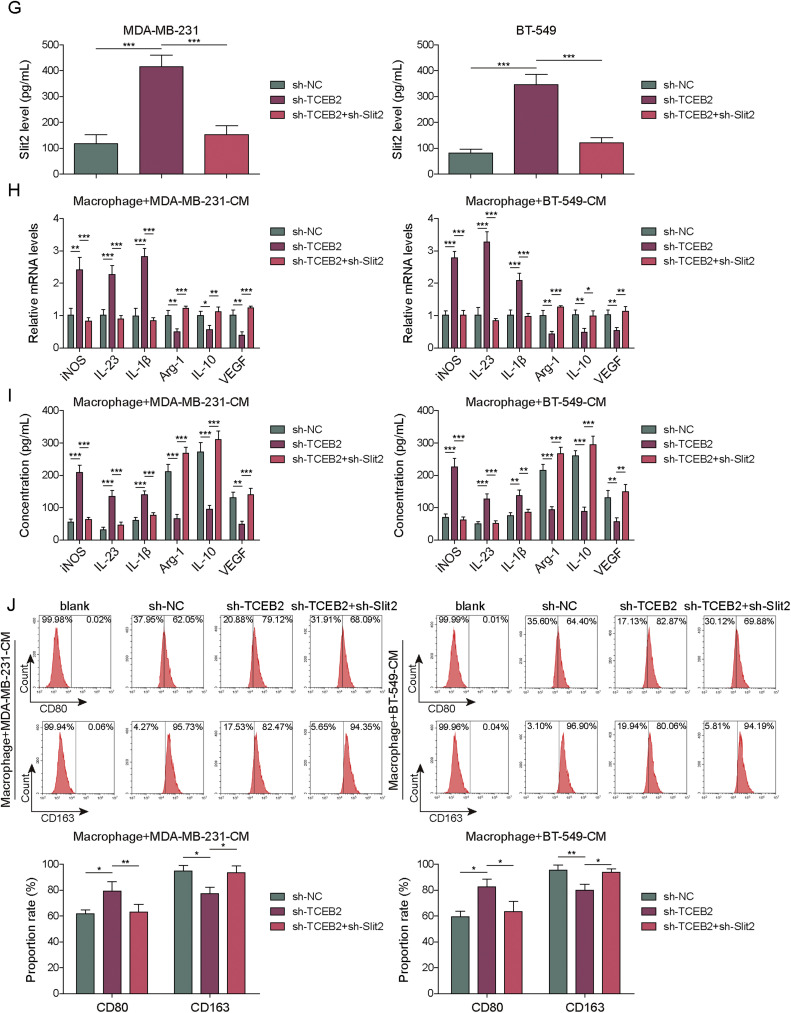
**5. TCEB2 promoted TNBC-induced M2 macrophage polarization by acting on Slit2**. (A-B) The mRNA and protein levels of Slit2 in MDA-MB-231 or BT-549 cells after sh-NC, sh-Slit2#1, sh-Slit2#2, or sh-Slit2#3 transfection were measured by qRT-PCR and western blot, respectively. Both TCEB2 knockdown and Slit2 knockdown were induced in MDA-MB-231 and BT-549 cells. (C-D) Cell viability and proliferation were analyzed by CCK8 assay and colony formation assay, respectively. (E-F) Cell migration and invasion were determined by wound healing assay and Transwell assay, respectively. (G) The level of Slit2 in conditioned media was detected using ELISA. (H-I) qRT-PCR and ELISA were adopted to measure the mRNA and secretion levels of iNOS, IL-23, IL-1β, Arg1, VEGF, and IL-10 in macrophages. (J) CD163 and CD80 levels in macrophages were analyzed by flow cytometry. The measurement data were presented as mean ± SD. *N* = 3. *p < 0.05, **p < 0.01, ***p < 0.001.

### TCEB2 knockdown inhibited TNBC-induced M2 macrophage polarization *in vivo*

**Fig. 6:**
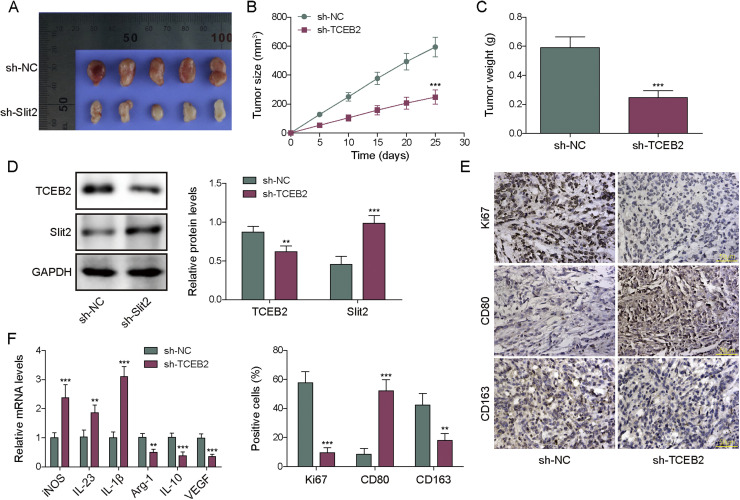
**TCEB2 knockdown inhibited TNBC-induced M2 macrophage polarization *in vivo***. The tumor implantation experiment was performed. (A-C) The size, volume, and weight of tumors were measured. (D) TCEB2 and Slit2 protein levels in tumor tissues were examined by western blot. (E) Ki67, CD80, and CD163 protein levels in tumor tissues were measured by IHC. (F) qRT-PCR was employed to determine the mRNA levels of iNOS, IL-23, IL-1β, Arg1, VEGF, and IL-10 in tumor tissues. The measurement data were presented as mean ± SD. *N* = 5. **p < 0.01, ***p < 0.001.

The tumor implantation experiment was used to verify the effect of TCEB2 on TNBC-induced M2 macrophage polarization *in vivo*. TCEB2 knockdown markedly inhibited tumor growth (Fig. 6**A-C**). Western blot results subsequently displayed that TCEB2 knockdown significantly reduced TCEB2 protein level but elevating Slit2 level in tumor tissues (Fig. 6**D**). In addition, TCEB2 knockdown markedly reduced the levels of ki67 (cell proliferating marker) and CD163 and increased CD80 level in tumor tissues (Fig. 6**E**). Furthermore, TCEB2 silencing significantly reduced the expressions of M2 macrophage cytokines (Arg1, VEGF and IL-10) and increased the expressions of M1 macrophage cytokines (iNOS, IL-23 and IL-1β) in tumor tissues (Fig. 6**F**). In summary, TCEB2 knockdown repressed TNBC-induced M2 macrophage polarization *in vivo*.

## Discussion

TNBC has the worst prognosis among all subtypes of breast cancer, which urges research efforts to find a new treatment scheme for TNBC [19]. It has been widely described that the immune system plays a significant role in cancer development [20]. Macrophage infiltration in tumors is closely related to the adverse clinical outcomes of TNBC [21]. The M1/M2 macrophage paradigm acts as a key part of tumor development. M1 macrophages have traditionally been thought to be anti-tumor, but M2-polarized macrophages contribute to a variety of pro-tumorigenic outcomes in cancer [22]. As reported, activated M2 macrophages are strongly associated with TNBC invasion and metastasis [23]. Repressing M2 macrophage polarization might be a viable TNBC treatment method. The current study found that TCEB2 promoted M2 macrophage polarization in TNBC by facilitating ubiquitination degradation of Slit2 through recruiting NEDD4. Notably, our study also revealed that TNBC-conditioned macrophages exhibited a mixed CD80^+^CD163^+^ phenotype, distinct from classical M1/M2 polarization states. These hybrid macrophages displayed significantly higher co-expression of both markers compared to undifferentiated M0 macrophages, suggesting TNBC actively reprograms macrophage plasticity rather than simply driving M2 polarization. This aligns with emerging evidence that TAMs often adopt transitional states co-expressing canonical polarization markers across cancers [24,25]. While CD80^+^CD163^+^ TAMs have been implicated in immune modulation and metastasis in other malignancies [26], their functional role in TNBC remains unexplored. We hypothesize these cells may balance pro-inflammatory (CD80) and immunosuppressive (CD163) functions to create a permissive microenvironment—a potential mechanism underlying TNBC’s resistance to current M1/M2-targeted therapies. Future studies should dissect whether this hybrid state correlates with patient outcomes or therapeutic responses.

TCEB2 is an 18 kDa ubiquitin-like protein that degrades target proteins through interaction with E3 ubiquitin ligase [27]. TCEB2 is a central component of an ubiquitin ligase complex for proteasomal degradation [28]. Notably, TCEB2 can achieve its role in diseases by recruiting E3 ubiquitin ligase [29]. The role of TCEB2 in malignancies has been studied. In renal cell carcinoma, Chen et al. confirmed that TCEB2 was a hub gene that might be involved in the malignant progression [30]. TCEB2 was also reported to be highly expressed in breast cancer [11]. However, the expression and role of TCEB2 in TNBC remain unknown. Our findings illustrated that TCEB2 was highly expressed in TNBC cells and tissues, and its knockdown suppressed TNBC cell proliferation and invasion as well as the ability of TNBC cells to induce M2 macrophage polarization. Collectively, TCEB2 upregulation promoted TNBC-induced M2 macrophage polarization.

Slit2 is a secreted protein and is considered an axon-guiding protein, which can regulate cancer cell invasion and metastasis by affecting multiple downstream pathways through binding to ROBO1 [31]. Slit2 is considered a tumor suppressor in multiple malignant tumors, including TNBC [32,33]. As revealed by Jiang et al., Slit2 overexpression impaired TNBC cell migration and invasion [33]. Consistently, our findings displayed that Slit2 was lowly expressed in TNBC. As widely reported, Slit2 activity is commonly regulated by ubiquitination modification in cancers [34,35]. In the current research, while TCEB2 knockdown significantly reduced Slit2 protein levels without altering its mRNA expression, this discrepancy suggested post-translational regulation mechanisms. This observation guided our subsequent investigation, confirming that TCEB2 mediated Slit2 ubiquitination degradation in TNBC, which has never been reported before. Additionally, Slit2 upregulation could repress breast cancer metastasis by activating M1 macrophage polarization [17]. Our findings revealed that the treatment of exogenous recombinant Slit2 protein significantly promoted M2 macrophage polarization *in vitro*. Notably, our functional experiments revealed that while TCEB2 knockdown suppressed TNBC-induced M2 polarization, this effect was substantially attenuated by concurrent Slit2 knockdown. This observation supports our proposed TCEB2/Slit2 regulatory axis rather than indicating Slit2′s predominance, as: (1) TCEB2 acts intracellularly to control Slit2 stability through ubiquitination, while (2) secreted Slit2 mediates intercellular communication with macrophages. This two-step mechanism explains why Slit2 manipulation showed more immediate effects on macrophage polarization—consistent with established roles of Slit2-ROBO signaling in regulating macrophage plasticity [36]. The novelty of our findings lies specifically in: Identifying TCEB2 as a previously unknown upstream regulator of Slit2 in TNBC; Revealing the ubiquitination-mediated mechanism controlling Slit2 abundance; Demonstrating how intracellular TNBC signaling (via TCEB2) can shape the immune microenvironment through Slit2-dependent macrophage reprogramming. Collectively, TCEB2 upregulation promoted TNBC-induced M2 macrophage polarization by mediating Slit2 ubiquitination degradation.

As reported, TCEB2 mediates the degradation of target proteins by interacting with E3 ubiquitination ligases [9]. NEDD4 is an E3 ubiquitin ligase that promotes tumor initiation, progression, migration, and resistance to anti-cancer treatments through ubiquitination of tumor suppressor genes [32]. E3 ligases are essential components of the ubiquitination cascade since they recognize and modify substrates with particular polyubiquitin chains [37]. As revealed by Wan et al., NEDD4 upregulation in breast cancer was associated with cancer progression and poor prognosis [38]. Moreover, NEDD4 silencing remarkably suppressed TNBC cell proliferation, migration, and mammosphere formation [39]. As reported, NEDD4 promotes the degradation of target proteins by facilitating K48- and K68-dependent polyubiquitination of proteins [40]. In mammalian cells, the most prevalent polyubiquitin chains are K48 and K63 [41]. The K48-linked chains act as proteasomal targeting signals, whereas the K63-linked chains are mostly involved in nonproteolytic activities, including inflammatory signaling and endocytosis [42]. K63 can also form ubiquitin polymeric chains and mediate the ubiquitin degradation of target proteins [43]. Notably, NEDD4 could enhance polyubiquitination by interacting with complexes dominated by TCEB2 [18]. Moreover, we discovered that NEDD4 was also regulated by Slit2 polyubiquitin with K48-linked and K63-linked chains. Herein, our results showed that TCEB2 mediated Slit2 K63 ubiquitination degradation in TNBC by interacting with NEDD4, which has never been reported before.

Our research proved that TCEB2 overexpression promoted M2 macrophage polarization in TNBC by mediating Slit2 K63 ubiquitination degradation, which interacts with NEDD4. These findings provide new targets for the diagnosis and treatment of TNBC.

## Ethics approval and consent to participate

This study was approved by the Ethics Committee of Hunan Provincial People’s Hospital and The first-affiliated hospital of Hunan normal university, and all subjects provided informed permission.

Hunan Provincial People’s Hospital and The first-affiliated hospital of Hunan normal university approved the animal studies.

## Consent for publication

The informed consent obtained from study participants

## Availability of data and material

All data generated or analysed during this study are included in this published article

## CRediT authorship contribution statement

**Li Liu:** Writing – original draft, Visualization, Validation, Methodology, Investigation, Formal analysis, Conceptualization. **Wang Xiao:** Writing – original draft, Visualization, Validation, Methodology, Investigation, Data curation, Conceptualization. **Jie Zeng:** Data curation. **Jianing Yi:** Formal analysis. **Haoli Gong:** Software, Resources. **Luyao Liu:** Writing – review & editing, Writing – original draft, Supervision, Project administration, Funding acquisition, Conceptualization.

## Declaration of competing interest

The authors declare that they have no known competing financial interests or personal relationships that could have appeared to influence the work reported in this paper.

## Appendix Supplementary materials

Image, image 1

Image, image 2

**Fig. S1. Characterization of macrophage polarization states**. (A) THP-1 cells were treated with 100 nM PMA for 48 h, and cell morphology was observed under a microscope. (B) Flow cytometry analysis was performed to detect CD80 and CD163 expression in the M0 group, M1 group (IFN-γ + LPS), M2 group (IL-4 + IL-13), and TNBC-induced macrophages. The measurement data were presented as mean ± SD. *N* = 3 **p* < 0.05, ***p* < 0.01, ****p* < 0.001. **Fig. S2.** M0 macrophages were treated with 10 μg/mL exogenous recombinant Slit2 protein for 2 h. (A) The level of Slit2 in conditioned media was detected using ELISA. (B-C) qRT-PCR and ELISA were adopted to measure the mRNA and secretion levels of iNOS, IL-23, IL-1β, Arg1, VEGF, and IL-10 in macrophages. (D) CD163 and CD80 levels in macrophages were analyzed by flow cytometry. The measurement data were presented as mean ± SD. *N* = 3. **p* < 0.05, ***p* < 0.01, ****p* < 0.001. Image, application 3
