# Supplementary figures and images for: TCEB2 promotes M2 polarization of macrophages in triple negative breast cancer by mediating ubiquitination degradation of Slit2 through recruiting NEDD4
Source: Transl Oncol. 2025 Sep 26;62:102536. doi: 10.1016/j.tranon.2025.102536 (PMC12508586; doi:10.1016/j.tranon.2025.102536)

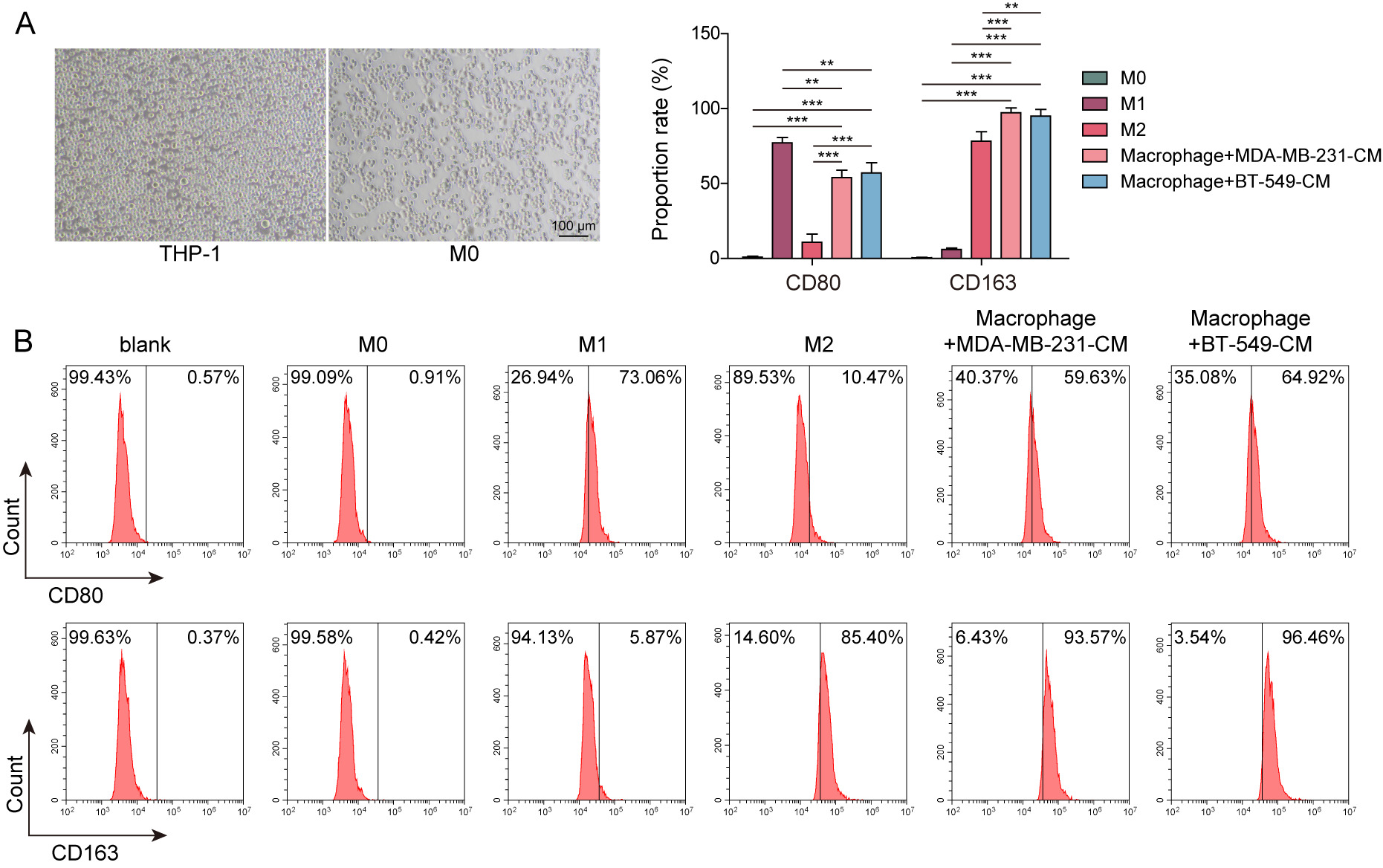

Supplement: Supplementary file 1 [file mmc1.jpg]

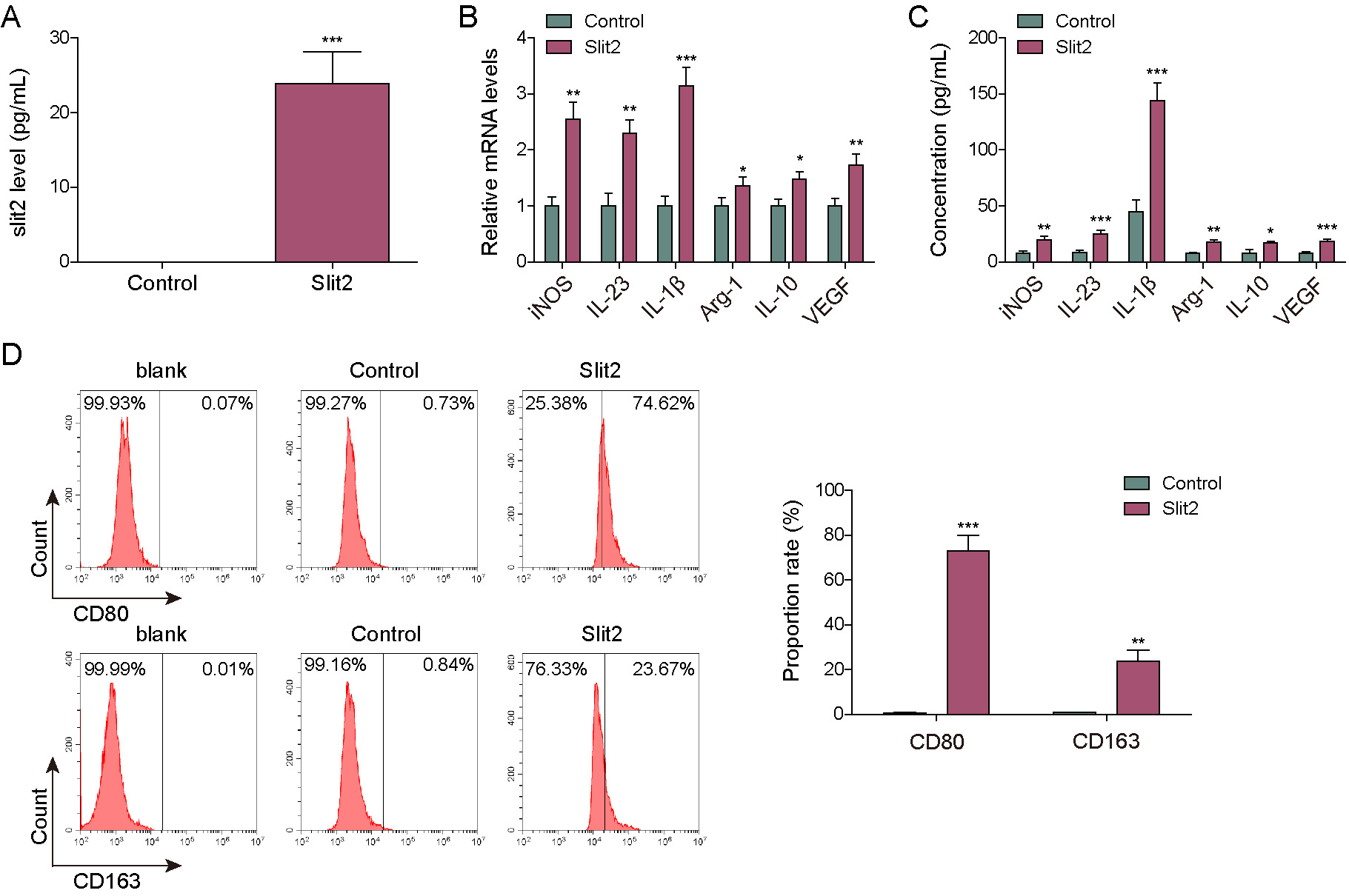

Supplement: Supplementary file 2 [file mmc2.jpg]
