# Supplementary material for: TCEB2 promotes M2 polarization of macrophages in triple negative breast cancer by mediating ubiquitination degradation of Slit2 through recruiting NEDD4
Source: Transl Oncol. 2025 Sep 26;62:102536. doi: 10.1016/j.tranon.2025.102536 (PMC12508586; doi:10.1016/j.tranon.2025.102536)
Supplement: Supplementary file 3 — Fig. S1. Characterization of macrophage polarization states. (A) THP-1 cells were treated with 100 nM PMA for 48 h, and cell morphology was observed under a microscope. (B) Flow cytometry analysis was performed to detect CD80 and CD163 expression in the M0 group, M1 group (IFN-γ + LPS), M2 group (IL-4 + IL-13), and TNBC-induced macrophages. The measurement data were presented as mean ± SD. N = 3 *p < 0.05, **p < 0.01, ***p < 0.001. Fig. S2. M0 macrophages were treated with 10 μg/mL exogenous recombinant Slit2 protein for 2 h. (A) The level of Slit2 in conditioned media was detected using ELISA. (B-C) qRT-PCR and ELISA were adopted to measure the mRNA and secretion levels of iNOS, IL-23, IL-1β, Arg1, VEGF, and IL-10 in macrophages. (D) CD163 and CD80 levels in macrophages were analyzed by flow cytometry. The measurement data were presented as mean ± SD. N = 3. *p < 0.05, **p < 0.01, ***p < 0.001. [file mmc3.docx]

**Table S1: Correlation of the expression levels of TCEB2, NEDD4, and Slit2 with Clinical Characteristics of Individual Patients with TNBC**

| Clinicopathologic features | | | Cases (n) | TCEB2 | | P-value | NEDD4 | | P-value | Slit2 | | P-value |
| --- | --- | --- | --- | --- | --- | --- | --- | --- | --- | --- | --- | --- |
|  |  |  |  | High (n) | Low (n) |  | High (n) | Low (n) |  | High (n) | Low (n) |  |
| Age | ＜35 | 14 | | 6 | 8 | 0.741 | 5 | 9 | 0.320 | 8 | 6 | 0.741 |
|  | ≥35 | 26 | | 14 | 12 |  | 15 | 11 |  | 12 | 14 |  |
| Menopausal Status | Yes | 8 | | 0 | 8 | **0.003**** | 1 | 7 | **0.043*** | 5 | 3 | 0.694 |
|  | No | 32 | | 20 | 12 |  | 19 | 13 |  | 15 | 17 |  |
| Lymph node status | Positive | 24 | | 16 | 8 | **0.022*** | 15 | 9 | 0.105 | 8 | 16 | **0.022*** |
|  | Negative | 16 | | 4 | 12 |  | 5 | 11 |  | 12 | 4 |  |
| TNM stage | I-II | 16 | | 4 | 12 | **0.022*** | 3 | 13 | **0.003**** | 14 | 2 | **0.0002***** |
|  | III-IV | 24 | | 16 | 8 |  | 17 | 7 |  | 6 | 18 |  |
